# Subchronic toxicity of herbal compound “Jiedu Huayu” granules in rats

**DOI:** 10.1186/s12906-017-1960-4

**Published:** 2017-09-06

**Authors:** Minggang Wang, Hua Qiu, Rongzhen Zhang, Fuli Long, Dewen Mao

**Affiliations:** Department of Liver Disease, The First Affiliated Hospital of Guangxi University of Traditional Chinese Medicine, Nanning, Guangxi Province 530023 China

**Keywords:** Subchronic toxicity, Chinese herbal compounds, “Jiedu Huayu” granules, Rat

## Abstract

**Background:**

“Jiedu Huayu” (JDHY) granules are traditional Chinese herbal compounds that have been used to treat severe liver injury for many years. The purpose of the current study is to evaluate the safety of JDHY granules.

**Methods:**

Subchronic toxicity was tested in male and female rats that were orally administered three different doses (80, 100, and 130 g/kg/d) of JDHY for 13 weeks. Clinical signs, bodyweight, food consumption, hematological and biochemical parameters, organ coefficients, and histological changes were observed during the study.

**Results:**

There were no significant changes in toxicity observed in either sex at any dose of JDHY granules treatment.

**Conclusions:**

These results suggest that repeated oral administration of JDHY granules at dosage levels of ≤130 g/kg/d can be considered safe.

## Background

Traditional Chinese herbal compounds have been widely used throughout history. They have been considered to be effective and have few side effects, particularly because of their natural origins [1]. The therapeutic effects of compounds depend on the components of each herb involved. In recent decades, the use of herbal compounds has rapidly increased. However, the safety of herbal compounds has been greatly debated [2].

“Jiedu Huayu” (JDHY) granules are traditional Chinese herbal compounds that are commonly used for the treatment of liver failure. These granules consist of six herbs: *Artemisia capillaries, Radix paeoniae rubrathe, Rheum officinale, Oldenlandia diffusa, Radix curcumae,* and *Acorus gramineus*. Previous clinical studies established that JDHY has hepato-protective effects in hepatitis B-related acute chronic liver failure patients, improved liver function, and reduced complications [3]. Moreover, animal experiments demonstrated that JDHY decreased the expression of Caspase-3 mRNA in a model of acute liver failure. This suggests that JDHY may be an effective therapy for treating hepatocyte-related apoptosis [4]. However, there is currently insufficient investigation into the toxicity of JDHY granules. In this study, we evaluate the toxicity and safety pharmacology of an oral administration of JDHY granules to rats for 13 weeks to assess an optimal dose for clinical use.

## Methods

### Experimental animals

Animal experiments were approved by The First Affiliated Hospital of Guangxi University of Traditional Chinese Medicine. Male and female Wistar rats weighing 100 g–120 g were purchased from the Animal Experimental Center of Guangxi Medical University (Nanning, China, License No. SCXK Gui 2009–0002). All rats were fed a Specific Pathogen-Free (SPF) diet.

### *Preparation* of JDHY granules

“Jiedu Huayu” (JDHY) granules were prepared in our laboratory from six herbs (Table 1): 667 g of *Artemisia capillaries*, 1000 g of *Radix paeoniae rubrathe*, 333 g of *Rheum officinale*, 500 g of *Oldenlandia diffusa*, 333 g of *Radix curcumae*, and 333 g of *Acorus gramineus*. The Chinese herbs were purchased from The First Affiliated Hospital of Guangxi University of Traditional Chinese Medicine. The herbal mixture underwent combined decoction. It was extracted twice in distilled water at 100 °C for 2 h. Following filtration, the resulting mixture was concentrated to a cream with a relative density between 1.05–1.10 (70 °C). Next, 60% ethanol was added for 24 h, and following filtration, the resulting mixture was then concentrated to a cream with a relative density of 1.30–1.35 (80 °C). Dextrins were added to make the final weight 1000 g, and the equivalent dose was 3.2 g/g (total raw materials/ weight after concentration). Quality control was performed as previously reported [5]. Prior to usage, JDHY granules were configured into three concentrations (6.5 g/ml, 5.0 g/ml, and 4.0 g/ml) by distilled water.

**Table 1:** Composition of JDHY granules

Components (Latin name)	Components (English name)	Family
*Artemisia capillaris Thunb*	*Capillary Wormwood Herb*	*Asteraceae*
*Paeonia veitchii Lynch*	*Radix paeoniae rubrathe*	*Ranunculaceae*
*Rheum palmatum L*	*Rheum officinale*	*Polygonaceae*
*Hedyotis diffusa Willd*	*Oldenlandia diffusa*	*Rubiaceae*
*Curcuma wenyujin Y.H. Chen et C.Ling*	*Radix Curcumae*	*Zingiberaceae*
*Acorus tatarinowii Schott*	*Acorus gramineus*	*Araceae*

### Experimental design

Forty-eight rats were randomized into four groups: control group (gavaged with distilled water,a low dose group (gavaged with JDHY granules 80 g/kg/d), a middle dose group (gavaged with JDHY granules 100 g/kg/d),and a high dose group (gavaged with JDHY granules 130 g/kg/d). Each experimental group consisted of 6 males and 6 females. Following an environmental adaptation period of 1 week, all rats were fed the SPF diet, and their initial weight was recorded. Rats were administered an amount of 20 ml/kg, which was adjusted once a week to accommodate changes in weight. Each group was gavaged for 6 days and rested for 1 day each week for a total duration of 13 weeks.

### Clinical observation, bodyweight, and food consumption

Clinical signs were observed twice daily for all rats. Clinical signs included changes in fur, mucous membrane, eyes, physical activity, behavior, fecal excretion, and mortality. Bodyweight and food consumption were recorded once a week [6].

### Hematology and serum biochemistry

Blood samples were collected and analyzed for white blood cell count (WBC), lymphocyte cell count (LYM), percentage of lymphocyte cell count (LYM%), reticulocyte cell count (RC), mononuclear cell count (MID), percentage of lymphocyte cell count (MID%), platelet count (PLT), granulocyte cell count (GRAN), percentage of granulocyte cell count (GRAN%), red blood cell count (RBC), hematocrit (HCT), corpuscular volume (MCV), concentration (HGB), hemoglobin (MCH), MCH concentration (MCHC). All samples were measured using automatic blood cell analyzer (mindray, China).

The following serum biochemistry parameters were also detected: aspartate aminotransferase (AST), alanine aminotransferase (ALT), alkaline phosphatase (ALP), total protein (TP), albumin (ALB), total bilirubin (TBIL), total cholesterol (CHOL), triglycerides (TG), glucose (GLU), blood urea nitrogen (BUN), creatinine (Cr), creatine kinase (CK), sodium (Na), potassium (K), and chloride (Cl). All samples were measured using automatic biochemical analyzer (mindray, China).

### Organ coefficient

We measured the absolute weight of heart, liver, spleen, lung, kidney, brain, thymus, renicapsule, testis (male), epididymis (male), uterus (female), and ovaries (female). Relative organ weight was calculated as a percentage of body weight [7].

### Histopathology

At the conclusion of the experiment, all animals were sacrificed using ether anesthesia. Tissues and organs were harvested and preserved in 10% formalin solution. All samples were sectioned and stained with hematoxylin and eosin [8]. We used a biological microscope and dp-20 digital imaging system (OLYMPUS, Japan) to conduct histopathological examination.

### Statistical analysis

Spss 18.0 software was used for statistical analysis. Data are presented as means ± standard deviation. Data were analyzed by one way analysis of variance (ANOVA) followed by the Student-Newman-Keuls test. Statistical significance was set at *P* < 0.05 as compared to the control group.

## Results

### Clinical signs, food consumption, and body weight

**Fig. 1:**
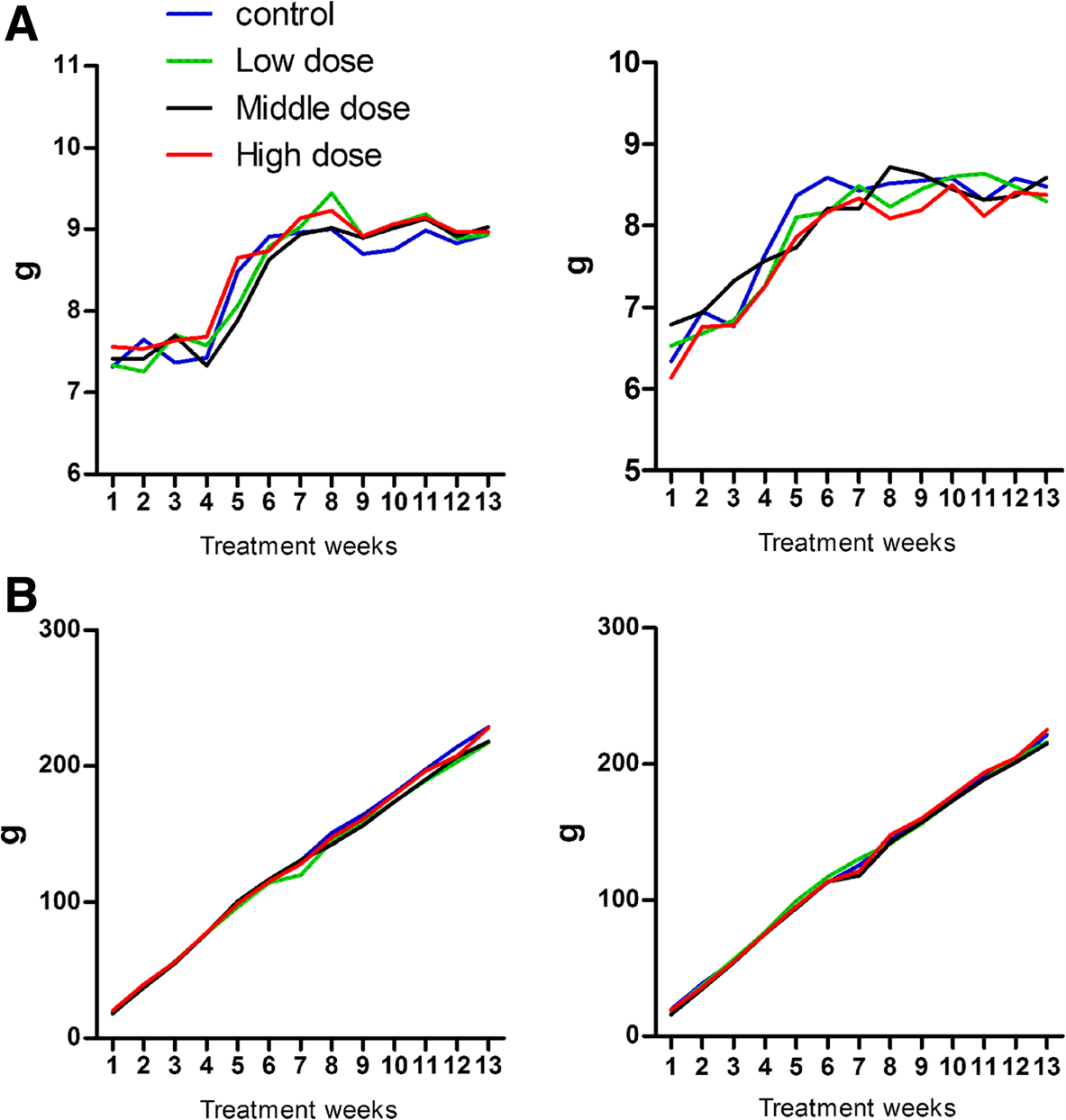
JDHF treatment does not alter food consumption or body weight. Wistar rats were treated with or without JDHF for 13 weeks. Food consumption (**a**) and body weight (**b**) were measured. Significance is indicated as *P* > 0.05

We did not observe any death or adverse clinical signs in the JDHY groups during the 13-week experiment. There were no statistically significant changes observed in food consumption (Tables 2 and 3, Fig. 1a) and body weight (Table 4 and 5, Fig. 1b) between control and JDHF groups.

**Table 2:** Food consumption of male rats treated with JDHY granules from 1 to 13 weeks

Dose(g/kg/d)	0	80	100	130
Males				
1 week	7.32 ± 0.67^a^	7.34 ± 0.39	7.42 ± 0.53	7.56 ± 0.44
2 week	7.65 ± 0.24	7.26 ± 0.48	7.42 ± 0.96	7.54 ± 0.86
3 week	7.37 ± 0.50	7.71 ± 0.38	7.70 ± 0.27	7.64 ± 0.37
4 week	7.43 ± 0.40	7.58 ± 0.20	7.33 ± 0.24	7.69 ± 0.18
5 week	8.48 ± 0.77	8.07 ± 0.59	7.89 ± 0.57	8.65 ± 0.88
6 week	8.91 ± 0.31	8.79 ± 0.40	8.63 ± 0.32	8.74 ± 0.26
7 week	8.96 ± 0.11	9.03 ± 0.20	8.94 ± 0.18	9.14 ± 0.19
8 week	9.00 ± 0.21	9.45 ± 0.20	9.02 ± 0.11	9.23 ± 0.34
9 week	8.70 ± 0.17	8.91 ± 0.16	8.90 ± 0.21	8.92 ± 0.19
10 week	8.75 ± 0.27	9.06 ± 0.27	9.02 ± 0.27	9.07 ± 0.24
11 week	8.99 ± 0.15	9.19 ± 0.18	9.13 ± 0.25	9.15 ± 0.24
12 week	8.83 ± 0.10	8.89 ± 0.21	8.92 ± 0.15	8.97 ± 0.13
13 week	8.94 ± 0.19	8.94 ± 0.11	9.03 ± 0.12	8.97 ± 0.12

All samples were collected from five rats in each group

^a^Data are given as mean ± SD

ANOVA analysis indicated no statistical significance *P* > 0.05

**Table 3:** Food consumption of Female rats treated with JDHY granules from 1 to 13 weeks

Dose (g/kg/d)	0	80	100	130
Females				
1 week	6.34 ± 0.26^a^	6.53 ± 0.17	6.79 ± 0.57	6.14 ± 0.62
2 week	6.95 ± 0.36	6.68 ± 0.41	6.94 ± 0.46	6.76 ± 0.49
3 week	6.77 ± 0.42	6.84 ± 0.58	7.32 ± 0.84	6.78 ± 0.49
4 week	7.64 ± 0.46	7.26 ± 0.30	7.57 ± 0.41	7.26 ± 0.20
5 week	8.37 ± 0.65	8.10 ± 0.51	7.73 ± 0.46	7.86 ± 0.51
6 week	8.59 ± 0.42	8.17 ± 0.42	8.21 ± 0.24	8.17 ± 0.61
7 week	8.43 ± 0.41	8.49 ± 0.41	8.21 ± 0.53	8.34 ± 0.31
8 week	8.52 ± 0.35	8.23 ± 0.20	8.72 ± 0.25	8.09 ± 0.37
9 week	8.55 ± 0.38	8.45 ± 0.38	8.63 ± 0.21	8.19 ± 0.20
10 week	8.58 ± 0.18	8.60 ± 0.19	8.45 ± 0.16	8.50 ± 0.16
11 week	8.31 ± 0.23	8.64 ± 0.32	8.32 ± 0.33	8.12 ± 0.16
12 week	8.58 ± 0.22	8.48 ± 0.24	8.36 ± 0.35	8.41 ± 0.08
13 week	8.48 ± 0.17	8.30 ± 0.13	8.59 ± 0.36	8.38 ± 0.35

All samples were collected from five rats in each group

^a^Data are given as mean ± SD

ANOVA analysis indicated no statistical significance *P* > 0.05

**Table 4:** Increase in body weight for male rats treated with JDHY granules from 1 to 13 weeks

Dose (g/kg/d)	0	80	100	130
Males				
0 week	122.3 ± 6.47^a^	122.2 ± 7.39	122.1 ± 7.24	118.7 ± 5.92
1 week	19.2 ± 5.10	17.6 ± 3.83	18.2 ± 4.81	19.8 ± 5.97
2 week	37.7 ± 8.65	37.7 ± 5.75	36.8 ± 8.30	39.3 ± 9.63
3 week	55.7 ± 9.00	56.3 ± 4.95	54.9 ± 7.97	55.9 ± 8.22
4 week	77.4 ± 9.50	77.1 ± 8.50	77.2 ± 12.31	77.6 ± 10.68
5 week	98.4 ± 11.56	96.4 ± 7.670	100.5 ± 10.74	98.9 ± 11.13
6 week	115.1 ± 15.83	114.4 ± 8.47	116.9 ± 15.10	115.1 ± 13.71
7 week	130.7 ± 15.61	119.9 ± 25.50	130.9 ± 15.41	128.1 ± 12.65
8 week	150.9 ± 19.60	144.5 ± 13.73	142.3 ± 24.12	147.4 ± 14.82
9 week	164.0 ± 17.64	158.1 ± 12.94	156.1 ± 23.93	160.9 ± 14.76
10 week	180.2 ± 15.36	173.9 ± 12.50	173.7 ± 22.06	178.8 ± 11.27
11 week	197.5 ± 14.54	189.3 ± 12.35	190.0 ± 22.71	196.5 ± 11.65
12 week	214.1 ± 16.34	203.2 ± 14.56	206.7 ± 22.71	207.3 ± 26.31
13 week	228.7 ± 16.53	217.3 ± 17.04	218.2 ± 21.72	228.2 ± 16.99

All samples were collected from five rats in each group

^a^Data are given as mean ± SD

ANOVA analysis indicated no statistical significance *P* > 0.05

**Table 5:** Increase in body weight for female rats treated with JDHY granules from 1 to 13 weeks

Dose (g/kg/d)	0	80	100	130
Females				
0 week	109.0 ± 5.59^a^	109.7 ± 7.70	110.3 ± 6.37	109.5 ± 6.12
1 week	19.5 ± 6.50	18.3 ± 4.93	15.8 ± 4.23	19.2 ± 5.06
2 week	38.7 ± 10.08	37.1 ± 7.84	34.7 ± 5.75	35.9 ± 7.75
3 week	54.9 ± 7.06	56.3 ± 6.63	54.1 ± 4.17	54.5 ± 8.63
4 week	75.4 ± 11.27	76.7 ± 11.98	75.3 ± 7.060	75.5 ± 11.17
5 week	95.0 ± 11.59	99.5 ± 10.85	94.2 ± 4.620	95.4 ± 13.66
6 week	113.1 ± 13.39	117.0 ± 13.33	113.5 ± 6.20	113.7 ± 16.78
7 week	125.9 ± 11.27	130.5 ± 14.74	118.2 ± 24.31	121.4 ± 26.69
8 week	144.1 ± 14.37	141.7 ± 24.10	142.4 ± 11.54	147.7 ± 20.69
9 week	157.6 ± 14.26	156.2 ± 23.75	157.1 ± 11.43	159.8 ± 20.17
10 week	174.7 ± 12.64	173.1 ± 21.59	173.0 ± 12.72	176.8 ± 20.38
11 week	192.4 ± 13.75	188.7 ± 22.59	188.5 ± 12.97	193.9 ± 19.52
12 week	202.1 ± 26.58	204.5 ± 21.15	201.2 ± 15.43	204.6 ± 29.79
13 week	221.7 ± 19.64	216.2 ± 23.10	215.1 ± 16.52	225.2 ± 20.75

All samples were collected from five rats in each group

^a^Data are given as mean ± SD

ANOVA analysis indicated no statistical significance *P* > 0.05

### Hematology and serum chemistry

The results of the hematological detection and serum chemistry examination are shown in Tables 6, 7, 8, and 9. There were no statistically significant changes in hematological detection and serum chemistry examination between the control and JDHY groups in male or female rats.

**Table 6:** Hematological data for male rats treated with JDHY granules for 13 weeks of treatment

Dose (g/kg/d)	0	80	100	130
Males				
WBC(10^9^/L)	7.91 ± 1.89^a^	7.65 ± 1.55	8.42 ± 1.74	8.03 ± 1.57
LYM(10^9^/L)	3.29 ± 1.13	3.77 ± 0.97	4.35 ± 1.98	4.20 ± 1.97
LYM%(%)	42.85 ± 13.78	49.99 ± 11.63	50.28 ± 19.21	50.44 ± 18.91
RC(%)	18.60 ± 3.27	18.50 ± 2.22	18.90 ± 3.21	18.30 ± 3.94
MID(10^9^/L)	0.92 ± 0.39	1.15 ± 0.34	1.29 ± 0.42	1.19 ± 0.44
MID(%)	11.36 ± 3.70	15.27 ± 4.89	15.83 ± 6.01	14.58 ± 4.58
PLT(10^9^/L)	513.40 ± 103.78	505.50 ± 135.51	491.00 ± 131.84	507.00 ± 115.41
GRAN(10^9^/L)	3.70 ± 1.58	2.73 ± 1.34	2.78 ± 1.12	2.64 ± 1.54
GRAN(%)	45.79 ± 13.22	34.74 ± 11.55	33.89 ± 14.58	34.98 ± 21.28
RBC(10^12^/L)	5.73 ± 1.63	6.06 ± 1.84	6.37 ± 1.46	6.31 ± 1.71
HCT(%)	42.45 ± 12.07	39.58 ± 15.08	45.74 ± 10.67	43.04 ± 15.63
MCV(fl)	66.76 ± 10.64	67.33 ± 7.83	67.91 ± 10.39	69.79 ± 8.39
HGB(g/L)	128.90 ± 24.14	131.20 ± 25.37	133.20 ± 23.90	126.70 ± 23.37
MCH(pg)	18.13 ± 1.77	18.79 ± 1.76	18.12 ± 1.09	18.48 ± 1.57
MCHC(g/L)	274.70 ± 9.35	280.00 ± 9.58	279.70 ± 9.31	282.70 ± 9.88

All samples were collected from five rats in each group

^a^Data are given as mean ± SD

ANOVA analysis indicated no statistical significance *P* > 0.05

**Table 7:** Hematological data for female rats treated with JDHY granules for 13 weeks of treatment

Dose (g/kg/d)	0	80	100	130
Females				
WBC(10^9^/L)	7.91 ± 1.89^a^	8.94 ± 1.25	7.44 ± 2.08	8.37 ± 2.10
LYM(10^9^/L)	3.42 ± 1.29	4.05 ± 0.97	3.58 ± 1.36	4.11 ± 1.44
LYM%(%)	46.59 ± 13.74	46.04 ± 11.71	47.17 ± 12.50	47.89 ± 11.92
RC(%)	16.40 ± 2.06	16.30 ± 2.11	16.80 ± 2.34	16.90 ± 2.07
MID(10^9^/L)	1.31 ± 0.61	1.27 ± 0.313	1.12 ± 0.24	1.36 ± 0.31
MID(%)	17.61 ± 5.48	14.19 ± 2.90	15.82 ± 4.41	16.73 ± 4.04
PLT(10^9^/L)	493.00 ± 122.40	517.30 ± 169.72	511.00 ± 180.39	514.10 ± 130.51
GRAN(10^9^/L)	2.69 ± 1.18	3.62 ± 1.45	2.74 ± 1.08	2.90 ± 0.90
GRAN(%)	35.80 ± 11.97	39.76 ± 11.84	37.01 ± 10.72	35.39 ± 10.62
RBC(10^12^/L)	5.55 ± 1.70	5.92 ± 1.79	5.77 ± 1.37	6.01 ± 1.28
HCT(%)	44.45 ± 12.36	43.62 ± 12.95	44.01 ± 13.52	59.29 ± 12.65
MCV(fl)	60.27 ± 10.80	64.38 ± 13.58	65.98 ± 13.23	69.79 ± 8.39
HGB(g/L)	132.10 ± 32.01	131.50 ± 23.08	135.50 ± 23.33	134.20 ± 28.76
MCH(pg)	18.70 ± 1.78	18.74 ± 1.44	18.50 ± 1.71	18.38 ± 1.23
MCHC(g/L)	280.20 ± 9.61	276.00 ± 8.61	274.20 ± 8.06	277.50 ± 6.46

All samples were collected from five rats in each group

^a^Data are given as mean ± SD

ANOVA analysis indicated no statistical significance *P* > 0.05

**Table 8:** Clinical chemistry data for male rats treated with JDHY granules for 13 weeks

Dose (g/kg/d)	0	80	100	130
Males				
AST(U/L)	217.31 ± 45.65	220.68 ± 66.49	219.30 ± 86.66	196.71 ± 77.69
ALT(U/L)	61.57 ± 9.09^a^	61.62 ± 10.59	56.86 ± 10.71	58.01 ± 12.24
ALP(U/L)	221.68 ± 88.98	239.23 ± 64.49	242.42 ± 81.93	266.23 ± 82.01
TP(g/L)	85.04 ± 4.67	82.7 ± 06.51	80.61 ± 6.84	86.03 ± 5.78
ALB(g/L)	42.04 ± 4.63	41.80 ± 4.78	40.84 ± 5.50	38.76 ± 5.31
TBIL(umol/L)	7.98 ± 2.34	8.30 ± 2.54	7.91 ± 2.30	7.86 ± 3.32
CHOL(mmol/L)	1.42 ± 0.45	1.64 ± 0.49	1.44 ± 0.46	1.53 ± 0.33
TG(mmol/L)	0.33 ± 0.11	0.35 ± 0.11	0.36 ± 0.08	0.38 ± 0.08
GLU(mmol/L)	7.06 ± 0.97	7.43 ± 0.74	7.33 ± 0.71	7.65 ± 0.91
BUN(mmol/L)	6.80 ± 1.11	6.78 ± 1.64	6.46 ± 1.41	6.47 ± 1.61
Cr(umol/L)	97.32 ± 15.24	97.65 ± 15.57	99.23 ± 15.67	98.54 ± 13.81
CK(U/L)	155.05 ± 25.48	147.16 ± 23.52	153.79 ± 26.00	150.48 ± 24.85
Na^+^(mmol/L)	150.29 ± 17.72	143.53 ± 15.43	141.36 ± 18.28	145.52 ± 21.69
K^+^(mmol/L)	4.59 ± 0.93	5.07 ± 1.31	4.96 ± 1.07	5.01 ± 0.84
Cl^−^(mmol/)	110.30 ± 6.30	105.50 ± 7.89	105.40 ± 6.93	108.00 ± 7.07

All samples were collected from five rats in each group

^a^Data are given as mean ± SD

ANOVA analysis indicated no statistical significance *P* > 0.05

**Table 9:** Clinical chemistry data for female rats treated with JDHY granules for 13 weeks

Dose (g/kg/d)	0	80	100	130
Females				
AST(U/L)	208.34 ± 47.04^a^	212.76 ± 79.64	217.31 ± 70.60	212.25 ± 89.47
ALT(U/L)	60.57 ± 10.31	60.10 ± 11.25	58.33 ± 11.02	61.53 ± 11.47
ALP(U/L)	237.88 ± 86.37	238.82 ± 78.02	227.31 ± 66.74	232.38 ± 85.91
TP(g/L)	84.12 ± 4.34	83.25 ± 8.49	80.47 ± 6.27	81.14 ± 5.41
ALB(g/L)	41.94 ± 5.24	42.61 ± 4.70	41.98 ± 5.82	41.43 ± 6.42
TBIL(umol/L)	8.23 ± 2.46	6.80 ± 2.88	8.59 ± 2.99	9.63 ± 3.09
CHOL(mmol/L)	1.49 ± 0.52	1.61 ± 0.49	1.58 ± 0.52	1.54 ± 0.37
TG(mmol/L)	0.32 ± 0.07	0.36 ± 0.11	0.37 ± 0.09	0.35 ± 0.07
GLU(mmol/L)	7.35 ± 0.72	7.20 ± 0.64	7.42 ± 0.61	7.57 ± 0.92
BUN(mmol/L)	6.51 ± 1.34	6.24 ± 1.36	6.35 ± 1.58	6.44 ± 1.18
Cr(umol/L)	98.63 ± 11.70	95.38 ± 15.75	96.72 ± 12.43	97.71 ± 12.70
CK(U/L)	155.77 ± 28.07	150.13 ± 26.80	149.74 ± 26.82	144.18 ± 28.09
Na^+^(mmol/L)	145.14 ± 17.59	148.75 ± 13.95	146.38 ± 11.86	144.94 ± 19.28
K^+^(mmol/L)	4.81 ± 0.92	5.34 ± 0.90	5.23 ± 1.12	4.93 ± 1.03
Cl^−^(mmol/L)	108.20 ± 5.95	105.80 ± 8.25	106.10 ± 8.55	107.30 ± 9.20

All samples were collected from five rats in each group

^a^Data are given as mean ± SD

ANOVA analysis indicated no statistical significance *P* > 0.05

### Organ coefficient

As shown in Tables 10 and 11, JDHY groups did not generate any statistically significant changes in organ coefficients in either male or female rats when compared with the control group.

**Table 10:** Organ coefficient for male rats treated with JDHY granules for 13 weeks

Dose (g/kg/d)	0	80	100	130
Males				
Heart	0.38 ± 0.051^a^	0.36 ± 0.051	0.36 ± 0.04	0.37 ± 0.06
Liver	3.44 ± 0.26	3.75 ± 0.57	3.51 ± 0.63	3.40 ± 0.53
Spleen	0.22 ± 0.05	0.25 ± 0.03	0.34 ± 0.03	0.22 ± 0.04
Lung	0.68 ± 0.14	0.62 ± 0.05	0.70 ± 0.05	0.71 ± 0.11
Kidney	0.62 ± 0.14	0.66 ± 0.06	0.71 ± 0.07	0.70 ± 0.10
Brain	0.48 ± 0.09	0.56 ± 0.09	0.52 ± 0.08	0.49 ± 0.09
Thymus	0.10 ± 0.04	0.09 ± 0.02	0.10 ± 0.03	0.10 ± 0.03
Renicapsule	0.02 ± 0.02	0.02 ± 0.01	0.02 ± 0.01	0.01 ± 0.03
Orchis	0.51 ± 0.16	0.49 ± 0.20	0.41 ± 0.14	0.49 ± 0.14
Epididymis	0.20 ± 0.09	0.27 ± 0.07	0.26 ± 0.05	0.24 ± 0.09

All samples were collected from five rats in each group

^a^Data are given as mean ± SD

ANOVA analysis indicated no statistical significance *P* > 0.05

**Table 11:** Organ coefficient for female rats treated with JDHY granules for 13 weeks

Dose (g/kg/d)	0	80	100	130
Females				
Heart	0.31 ± 0.03^a^	0.28 ± 0.03	0.27 ± 0.04	0.33 ± 0.09
Liver	3.27 ± 0.44	3.27 ± 0.45	3.18 ± 0.67	3.26 ± 0.38
Spleen	0.30 ± 0.12	0.34 ± 0.11	0.35 ± 0.12	0.35 ± 0.11
Lung	0.55 ± 0.11	0.61 ± 0.26	0.63 ± 0.28	0.73 ± 0.37
Kidney	0.61 ± 0.08	0.64 ± 0.08	0.64 ± 0.08	0.60 ± 0.07
Brain	0.51 ± 0.07	0.47 ± 0.04	0.53 ± 0.09	0.45 ± 0.07
Thymus	0.17 ± 0.08	0.22 ± 0.06	0.22 ± 0.08	0.18 ± 0.11
Renicapsule	0.02 ± 0.006	0.02 ± 0.004	0.02 ± 0.005	0.02 ± 0.006
Uterus	0.23 ± 0.06	0.24 ± 0.03	0.25 ± 0.12	0.21 ± 0.05
Oarium	0.06 ± 0.01	0.07 ± 0.02	0.05 ± 0.01	0.06 ± 0.02

All samples were collected from five rats in each group

^a^Data are given as mean ± SD

ANOVA analysis indicated no statistical significance *P* > 0.05

### Histopathology

**Fig. 2:**
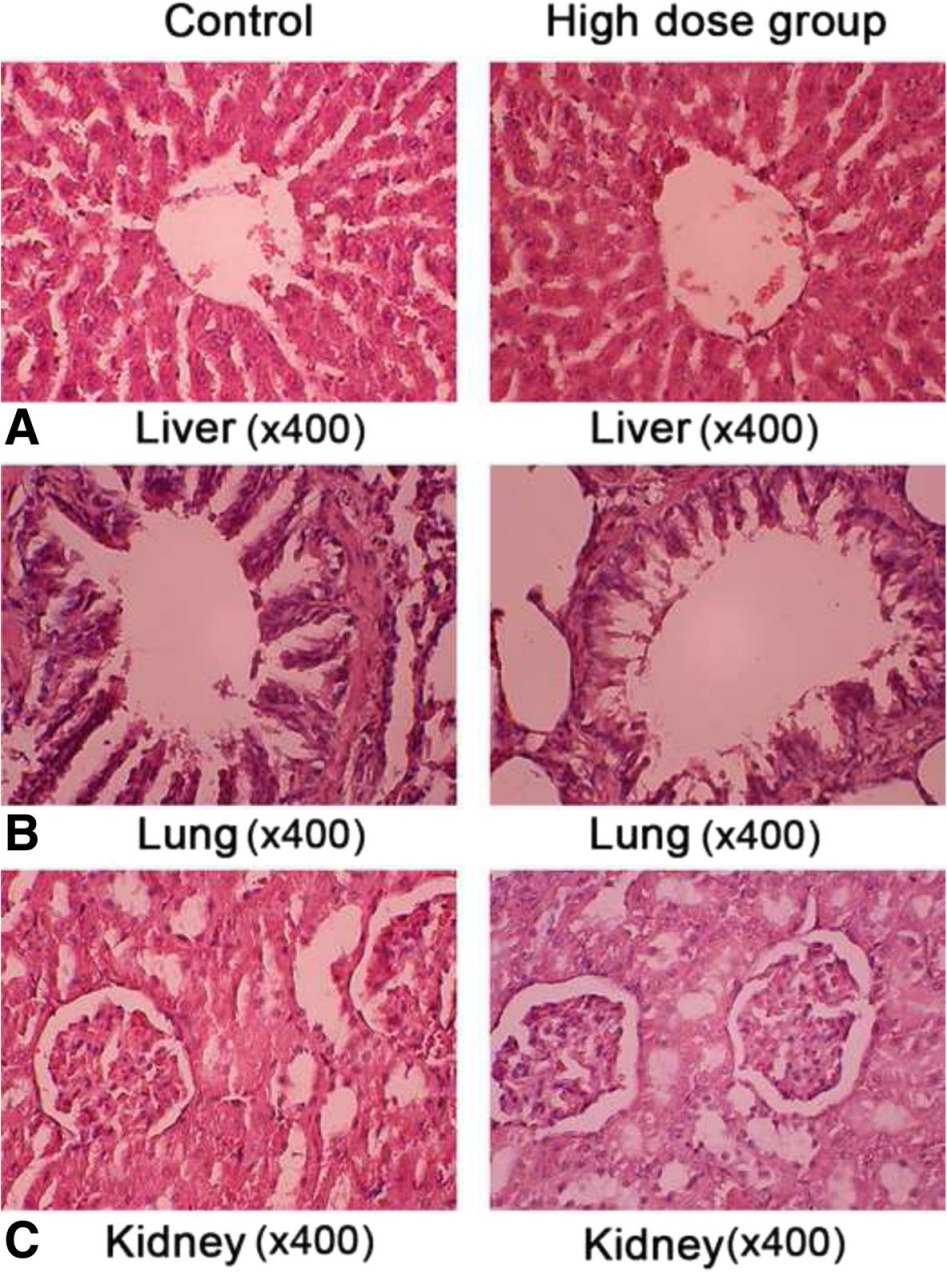
JDHF treatment maintains normal structure of liver, lung, and kidney. Representative images of H&E-stained sections of liver (**a**), lung (**b**), and kidney (**c**) from Wistar rats treated with or without JDHF are shown. Images were taken at a magnification of 400×

Following a 13-week treatment with JDHY granules, there were no treatment-related changes in histopathology detected between the control and JDHY groups. In fact, histopathological analysis of liver, lung, and kidney showed normal structure in all groups (Fig. 2).

## Discussion

Changes in food consumption and body weight are considered to be an indicator of organ toxicity [9]. In our 13-week study, we observed no deaths or abnormal behavior performance in all groups. While increases in food consumption and body weight were observed for all groups, there were no statistically significant differences between those given different doses of JDHF and the control group. Therefore, different doses of JDHY granules below or equal to 130 g/kg/d had no side effects on growth in male or female rats.

The circulatory system is essential for body function. Such functions include providing nutrients to different organs and carrying cytokine factors for immune reaction. Thus, the circulatory system is sensitive to toxicity of drugs [8]. In this study, we did not observe any statistically significant hematological differences between the JDHF groups and the control group. Levels of WBC, PLT, and HGB were in the normal range. Serum ALT, AST, BUN, and Cr can reflect liver and kidney function and are markers of liver and kidney damage [10, 11]. There were no statistically significant differences in these serum parameter levels between the JDHF groups and the control group. Thus, JDHY granules did not have a damaging effect on liver and kidney function.

Organ coefficients can provide direct evidence for pathological changes and can indicate organs influenced by drugs. In our study, analysis of organ weights resulted in no statistically significant changes between the JDHF groups and the control group. Furthermore, we used histopathological analysis to determine any pathological changes in liver, kidney, or lung because these organs play a significant role in the accumulation of drugs. We observed no histopathological malignancies in liver, kidney, and lung at a high dose of JDHY group. These results indicate that JDHY granules have no chronic toxicity for critical organs.

## Conclusion

Oral administration of JDHY granules to rats for 13 weeks did not have adverse side effects in both male and female rats of a dose up to 130 g/kg/d. Thus, no toxic effects were produced from treatment with JDHY granules.

## Abbreviations

ALB: Albumin
ALP: Alkaline phosphatase
ALT: Alanine aminotransferase
AST: Aspartate aminotransferase
BUN: Blood urea nitrogen
CHOL: Total cholesterol
CK: Creatine kinase
Cl: Chloride
Cr: Creatinine
GLU: Glucose
GRAN: Granulocyte cell count
GRAN%: Percentage of granulocyte cell count
HCT: Hematocrit
HGB: Concentration
JDHY: Jiedu Huayu granules
K: Potassium
LYM: Lymphocyte cell count
LYM%: Percentage of lymphocyte cell count
MCH: Hemoglobin
MCHC: MCH concentration
MCV: Corpuscular volume
MID: Mononuclear cell count
MID%: Percentage of lymphocyte cell count
Na: Sodium
PLT: Platelet count
RBC: Red blood cell count
RC: Reticulocyte cell count
TBIL: Total bilirubin
TG: Triglycerides
TP: Total protein
WBC: White blood cell count

## References

[1] Tang JL, Liu BY, Ma KW (2008). Traditional Chinese medicine. Lancet.

[2] Firenzuoli F, Gori L (2007). Herbal medicine today: clinical and research issues. Evid Based Complement Alternat Med.

[3] Na Wang SW, Tang N, Long F, Mao D (2014). Clinical study of leading integrated Chinese and Western medicine treatment of chronic on acute liver failure in the Jieduhuayu granule. Chinese Journal of Integrated Traditional and Western Medicine on Liver Diseases.

[4] Dewen Mao YC, Yu J, Huang G, Wang L, Long F (2009). Effect of Jieduhuayu Granule on the Expression ofCaspase-3mRNA in Liver Cells of Acute Liver Failure Mice. Lishizhenmedicine and Materiamedica Research.

[5] Dewen Mao HQ, Nong T, Xia X, Long F, Zhang R (2014). Research on Quality Criterion for Jieduhuayu Granule. Guangxi Journal of Traditional Chinese Medicine.

[6] Lee MY, Seo CS, Kim YB, Shin HK (2015). Safety assessment of Guibi-tang: Subchronic toxicity study in Crl:CD SD rats. Regulatory toxicology and pharmacology : RTP.

[7] Lee YH, Kim D, Lee MJ, Kim MJ, Jang HS, Park SH, Lee JM, Lee HY, Han BS, Son WC (2014). Subchronic toxicity study of Coptidis rhizoma in rats. J Ethnopharmacol.

[8] Lee MY, Seo CS, Shin IS, Kim YB, Kim JH, Shin HK (2013). Evaluation of oral subchronic toxicity of soshiho-tang water extract: the traditional herbal formula in rats. Evid Based Complement Alternat Med.

[9] Lee MY, Seo CS, Cha SW, Shin HK (2014). Safety assessment of So-cheong-ryong-tang: subchronic toxicity study in Crl:CD Sprague Dawley rats. Mol Med Rep.

[10] Ozer J, Ratner M, Shaw M, Bailey W, Schomaker S (2008). The current state of serum biomarkers of hepatotoxicity. Toxicology.

[11] Fuchs TC, Frick K, Emde B, Czasch S, von Landenberg F, Hewitt P (2012). Evaluation of novel acute urinary rat kidney toxicity biomarker for subacute toxicity studies in preclinical trials. Toxicol Pathol.

